# Surgical Principles and Minor Surgery

**Published:** 1881-06

**Authors:** 


SURGICAL PRINCIPLES AND MINOR SURGERY. By J. G. Gilcheist, M.D., pp. 205. Chicago, Duncan Brothers, 1881.

Dr. Gilcheist is already well and favorably known to the profession as the author of an excellent treatise on "Surgical Therapeutics." The volume now before us is a complete work on minor surgery, and is the second in a series of four, promised the profession by Prof. Gilcheist. The completed series will be: (1) "Minor Surgery," (2) "Surgical Therapeutics," (3) Surgical Emergencies," and (4) "Surgical Operations."

True to his colors, Dr. G. omits all mention of those worse than useless, allopathic expedients, blisters, hypodermic injections, etc. Yet we must take exception to our author's recommendation of blood-letting "in profound coma;" he thinks, "the chief difficulty that the mere therapeutist will encounter in cases of pure coma or asphyxia, will be the suspension of all the organic functions to such an extent that the absorption of the remedies given, by which alone their action can be developed, can not occur." We do not agree to this, nor have we ever seen such conditions. From our limited experience, we would say that in cases where the *simillimum* remedy fails, the knife will be useless.

There are excellent chapters on vaccination, catheterism, splints, bandages, etc., with illustrations. Dr. Gilcheist's work is fully equal to either Sargent's, Hill's or Leonard's.

